# Thyroid Lobectomy Under Local Anesthesia Before Lung Transplantation Owing to Diffuse Panbronchiolitis: A Case Report

**DOI:** 10.7759/cureus.54960

**Published:** 2024-02-26

**Authors:** Tsuyoshi Kojima, Yo Kishimoto, Keigo Honda, Atsushi Suehiro, Koichi Omori

**Affiliations:** 1 Department of Otolaryngology-Head and Neck Surgery, Graduate School of Medicine, Kyoto University, Kyoto, JPN

**Keywords:** thyroid cancer, diffuse panbronchiolitis, local anesthesia, lobectomy, thyroid

## Abstract

We herein report a case of thyroid lobectomy performed under local anesthesia for thyroid cancer in a patient who was at a high risk for general anesthesia due to diffuse panbronchiolitis. Although thyroid surgery has been performed in the past under local anesthesia in low-risk patients, thyroid surgery is now rarely performed under local anesthesia. If they are performed, thyroid surgery under local anesthesia is usually performed under monitored anesthesia care; sedation is considered safe and does not cause discomfort to patients. The present patient's respiratory function was poor, raising concerns that once intubated, extubation may not be possible because of the potential deterioration of respiratory function caused by the suppression of spontaneous breathing. Therefore, sedatives were avoided to maintain spontaneous breathing as much as possible. In such high-risk patients, additional care is required to ensure that the procedure is completed with minimal discomfort from pain or dyspnea. Maintaining a slightly upright position and raising the anesthesia screen are necessary to ensure a large space in front of the patient's face. In addition, an appropriate skin incision should be made to obtain a wide field of vision, and local anesthetic injections should be administered frequently to preemptively counter pain. Atropine sulfate was administered to reduce salivation and swallowing. Energy devices effectively reduced blood loss and operative time. Controlling intraoperative pain and bleeding is important, and the methods and techniques are also beneficial in surgery under general anesthesia.

## Introduction

With improvements in techniques and safety, many surgical procedures are now performed under general anesthesia. Similarly, there was a time when thyroid surgery was sometimes performed under local anesthesia; however, it is currently performed under general anesthesia [1]. In head and neck surgery, the surgical field is deep, and the field of vision is often small. Consequently, surgery under general anesthesia is the standard approach, considering the patient's discomfort during the operation due to poor visual field resulting from body movements and bleeding. This choice is particularly relevant for surgeries with the surgical field around the face, where sensory organs are concentrated. However, it is crucial to control intraoperative pain and bleeding to avoid discomfort in patients under local anesthesia, and the methods and techniques used for managing these issues are also beneficial in surgery under general anesthesia. Herein, we describe the case of a patient who was diagnosed with thyroid cancer and underwent thyroid lobectomy under local anesthesia while considering lung transplantation owing to respiratory failure caused by diffuse panbronchiolitis.

## Case presentation

The patient was a 30-year-old woman. Eleven years prior to her initial visit, she was diagnosed with diffuse panbronchiolitis by a respiratory physician after an abnormal shadow in the thorax was detected on physical examination. Thereafter, she was treated with clarithromycin, ambroxol hydrochloride, and tiotropium bromide hydrate inhalation, without any problems in her daily life. Two years before surgery, she experienced respiratory distress and was diagnosed with type 2 respiratory failure (SpO2: 60%; PaCo2: 56.1 mmHg). Subsequently, she was admitted to the hospital four to five times a year. She visited our lung transplantation surgery department and was considered for lung transplantation.

Six months before surgery, computed tomography (CT) revealed an incidental nodule in the left lobe of the thyroid gland, which was suspected to be a papillary carcinoma and diagnosed as papillary thyroid carcinoma (cT2N0M0, stage I) by ultrasound-guided fine-needle aspiration cytology at the otorhinolaryngology department of the previous hospital. Rendering the patient needed cancer-free through surgical intervention was a prerequisite for their eligibility for a lung transplant.

Based on her respiratory condition, she was referred to our hospital for perioperative management, including intensive care. The respiratory physician advised that there was a risk of difficult extubation associated with general anesthesia, and we decided to perform surgery under local anesthesia using a system that would allow intraoperative transfer to general anesthesia in case of an emergency.

Preoperative examination

**Figure 1:**
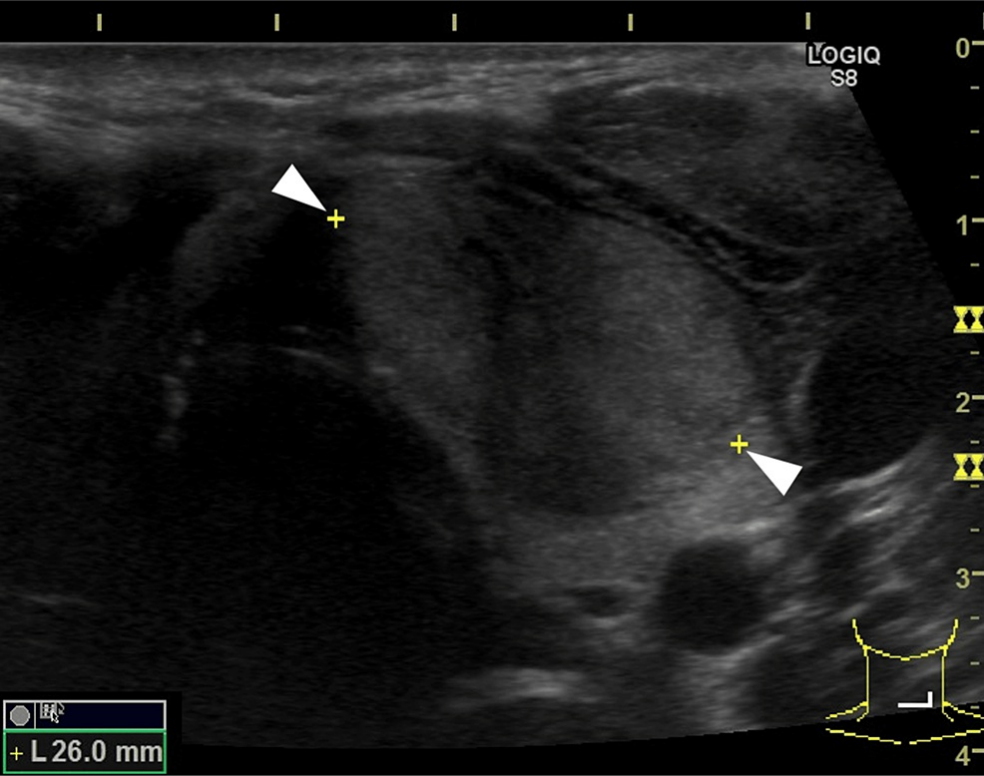
Ultrasound Ultrasound: 26×20×16 mm hypoechoic mass in the left lobe

**Figure 2:**
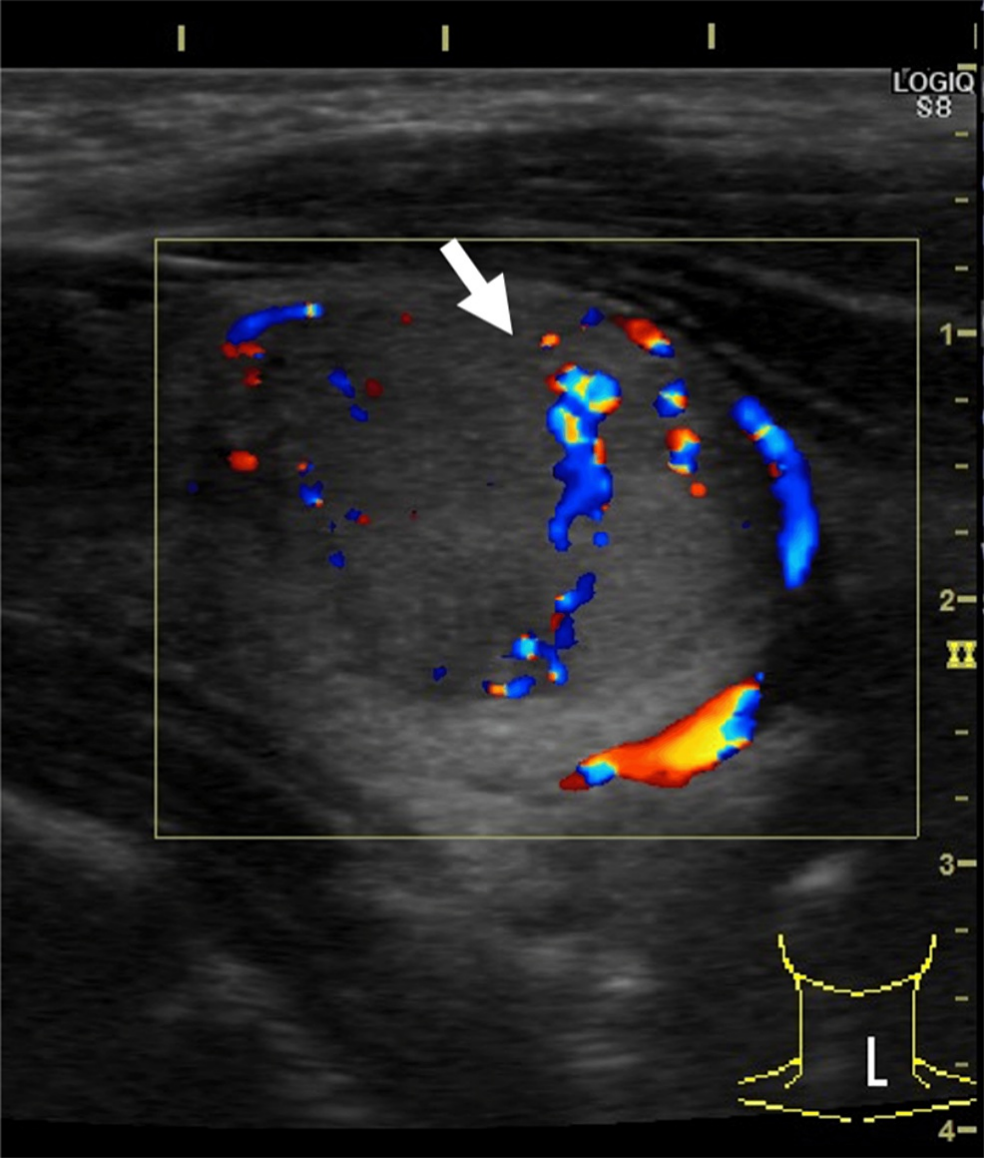
Ultrasound Blood flow inside the tumor and well-defined borders

Respiratory function tests showed a vital capacity (%) of 60.3%, forced expiratory volume in the first second (FEV1) of 740 mL, and FEV1% of 33.3%. The ultrasound showed a 26×20×16 mm hypoechoic mass in the left lobe with strong internal echoes and well-defined borders (Figure 1 and Figure 2).

**Figure 3:**
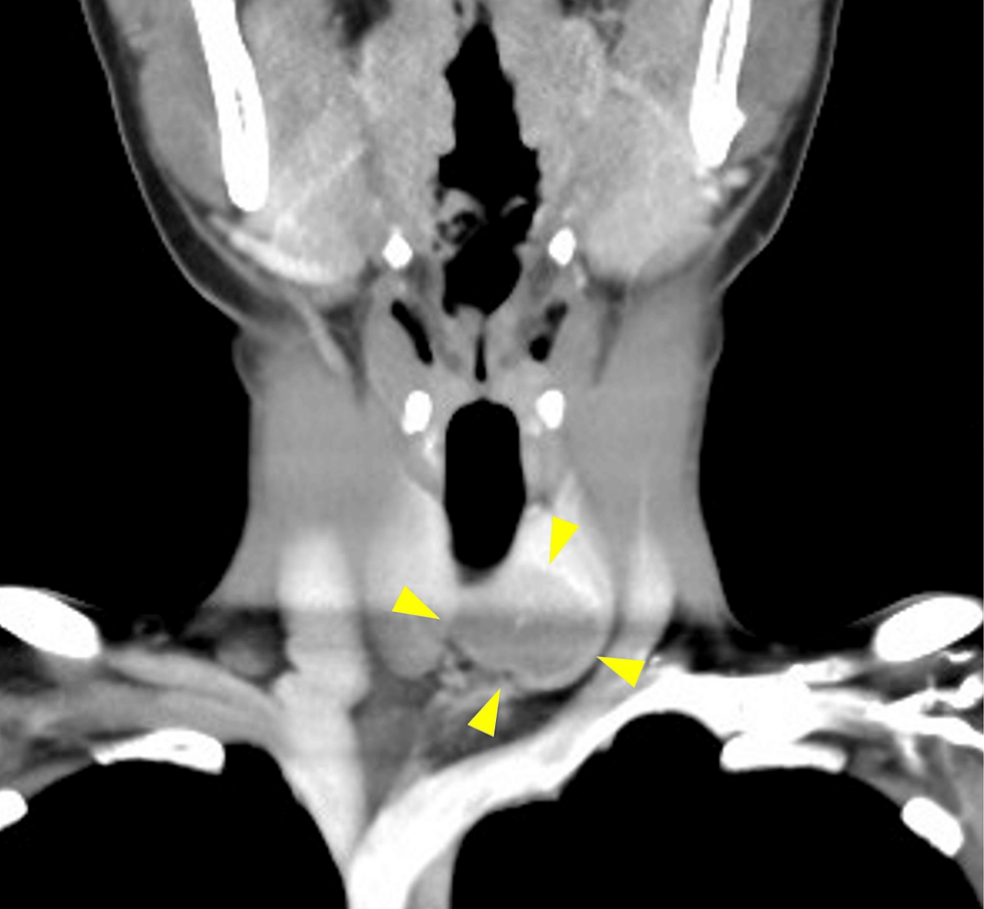
CT (coronal) A low-density area from the left lobe to the isthmus CT: computed tomography

**Figure 4:**
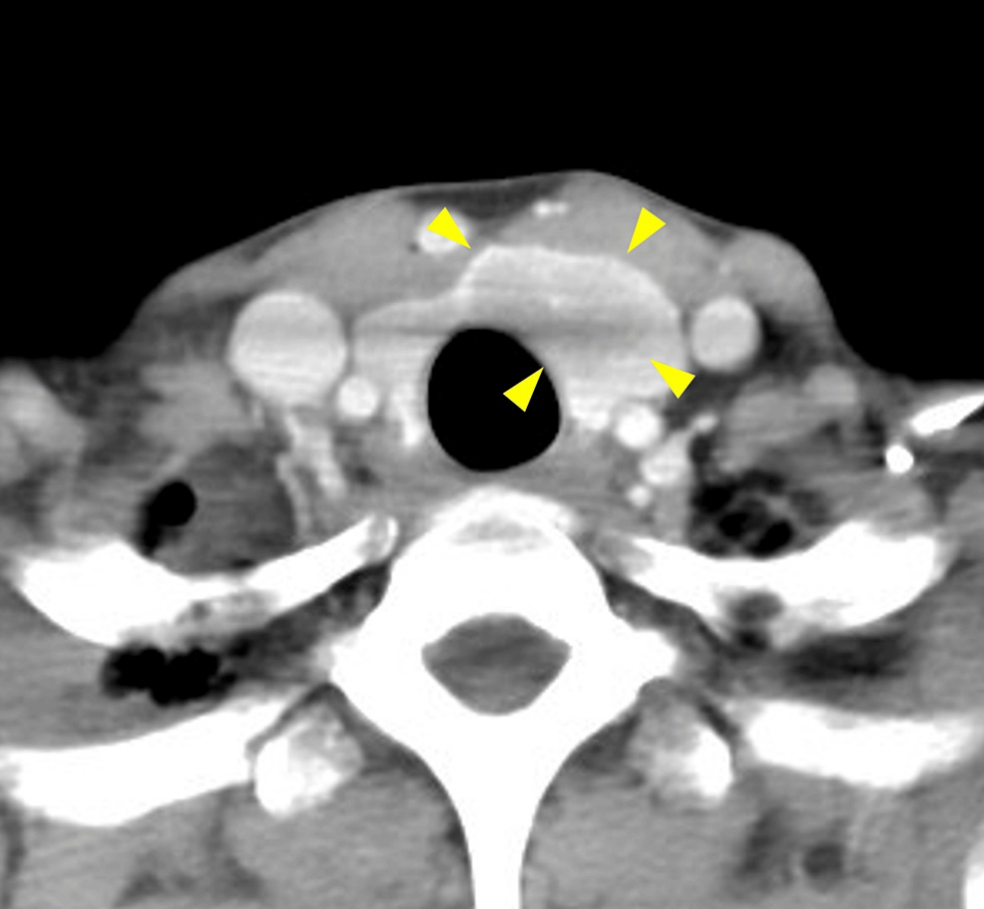
CT (axial) A low-density area from the left lobe to the isthmus CT: computed tomography

CT showed a low-density area from the left lobe to the isthmus and some extension beyond the midline to the right side (Figure 3 and Figure 4).

Magnetic resonance imaging (MRI) showed no obvious tracheal invasion. A 2-cm, well-defined mobile mass was palpated in the left thyroid lobe. Vocal cord paralysis was not observed.

Surgical findings

**Figure 5:**
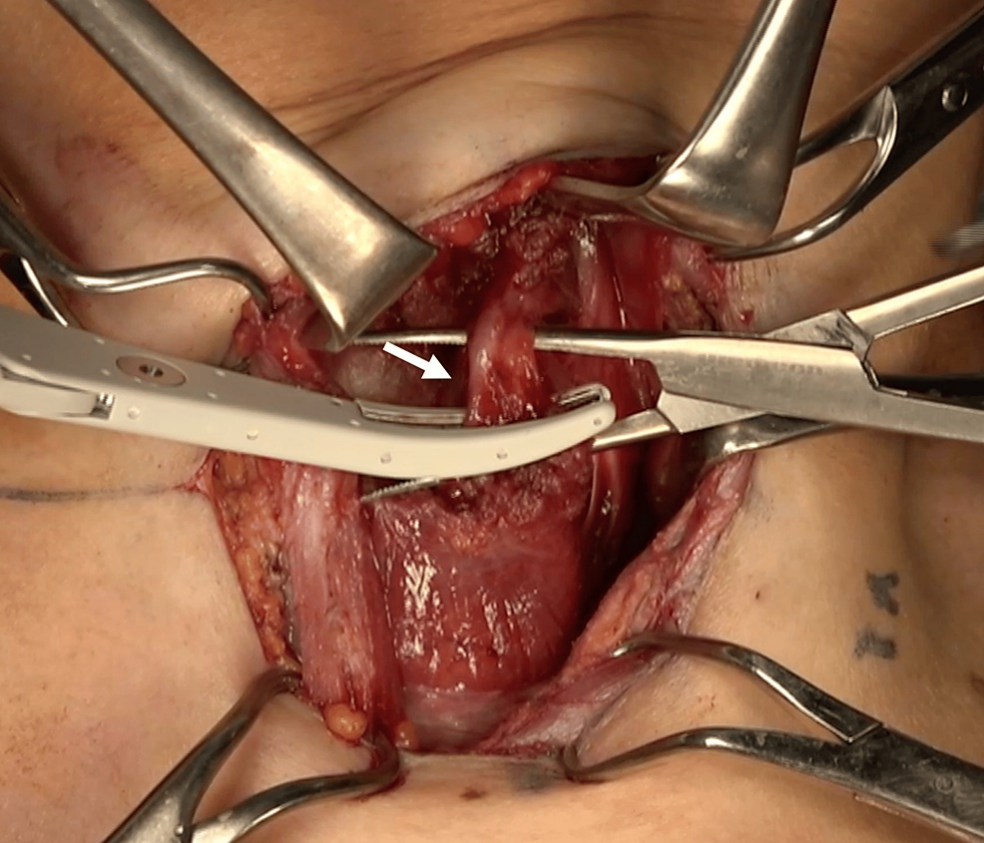
The inferior thyroid artery Hemostasized with a vessel-sealing device

Before positioning for surgery, the neck incision was marked, and local anesthesia was administered with 0.5% xylocaine with 1:100,000 epinephrine. Anesthesia was administered to the upper and lower skin flaps with 9 mL and the planned incision site with 6 mL. Additional doses of local anesthetic were administered intraoperatively with another 15 mL and as needed before dissecting the muscle and other tissues to ensure that the patient did not feel pain. The patient was placed in the supine position with a lighter-than-usual neck extension, and intravenous atropine sulfate was administered to reduce intraoperative salivary aspiration; antibiotics were administered preoperatively. The anesthesia screen was positioned sufficiently high to allow a large space in front of the face. A slightly larger 6.5-cm collared skin incision was made because no muscle relaxants were used and traction of the sternocleidomastoid and other muscles was expected to be difficult. Skin elevation was done upward to where the notch of the thyroid cartilage touches and downward to the clavicle, and local anesthesia was injected into that area prior to surgery. The sternocleidomastoid and sternohyoid muscles are dissected and retracted laterally and medially to secure the view. The sternothyroid muscle is excised above and below the thyroid gland and attached to the thyroid gland for resection. The superior thyroid arteriovenous and middle thyroid veins were hemostasized using a vessel-sealing device (Figure 5).

The thyroid gland was rotated, and the recurrent nerve was confirmed and preserved. As respiratory distress associated with tracheal compression was anticipated with thyroid gland transposition, the gland was gradually transposed while dissecting the surrounding area. The tumor was 2.5 cm in size, showing good mobility and no invasion into the surrounding area on visual examination. The inferior thyroid artery was hemostasized with a vessel-sealing device and ligation. 

A D1a dissection was performed. The use of monopolar devices was avoided as much as possible due to the large movement of the trachea with respiration. The right side of the isthmus was coagulated and cut using a vessel-sealing device, and the left lobe was removed. The patient did not complain of pain during the surgery, and oxygen was administered during the procedure because the SpO2 occasionally dropped to <90%. In addition, the bed was tilted approximately 20° so that the patient could be placed head-up during the surgery to alleviate respiratory distress. The operative time was 114 minutes, including interruptions, and the blood loss was 10 mL. The total amount of 0.5% xylocaine was 30 mL, less than the maximum amount.

Postoperatively, oxygen was continued at 2 L until the following morning, and her SpO2 remained at approximately 95%, the same as before surgery, with no complaints of respiratory distress. No vocal cord paralysis was observed. No abnormalities were shown at the wound site. Pain was controlled by administering acetaminophen three times a day until the second postoperative day, and no opioids were used. The wound drain was removed on the third postoperative day. The patient was discharged on the fifth postoperative day. Her temperature had remained in the normal range since admission, and she was discharged from the hospital without any major changes. The pathological results showed papillary thyroid carcinoma (follicular variant).

## Discussion

Until the 1980s, thyroid surgery was often performed under local anesthesia. However, thyroid surgery is currently rarely performed under local anesthesia [2]. Nevertheless, there have been still reports of thyroid surgeries performed under local anesthesia and monitored anesthesia care. This sedation management method uses local anesthesia and analgesic and sedative drugs to support the respiratory and circulatory functions to ensure patient safety and comfort [3,4]. It has the advantage of rapid postoperative recovery compared to general anesthesia and can be performed as an outpatient surgery. Additionally, when experienced surgeons perform this surgery, patient satisfaction is high, and medical costs are reduced [5]. While such surgery has been occasionally performed in high-risk cases [6], the associated benefits are usually reserved for low-risk patients, and for high-risk patients, the standard approach is surgery under general anesthesia.

The present patient's respiratory function was so poor that the respiratory physician had concerns that extubation may not have been possible once the patient was intubated and placed on a ventilator. Although clear criteria are difficult to define, FEV1 of less than 1 L is considered unsuitable for general anesthesia due to the high risk of difficult extubation and postoperative complications. Lung transplantation is the only way to improve respiratory function, and a prerequisite for transplantation is that the patient must be cancer-free, and surgery under local anesthesia is essential. Thyroid surgery under local anesthesia is typically performed under sedation. However, in this case, only injectable local anesthesia was used under the supervision of an anesthesiologist, and surgery was completed while maintaining spontaneous breathing. While nerve blocks and opioid analgesics are reportedly effective in controlling pain [4,6], pain control could be achieved through local anesthesia with a xylocaine injection. However, the patient experienced dyspnea due to the pressure caused by manipulation of the trachea when the thyroid gland was detached, and the surgery was interrupted several times. Had we been able to use sedation to relieve the patient's discomfort caused by the pressure sensation, the surgery might have progressed more smoothly. 

An important aspect of surgery under local anesthesia is to eliminate as much patient discomfort as possible. Surgery in such cases should be performed with the patient's head up and the anesthesia screen as high as possible to reduce breathing-related stress related to the patient's breathing. The skin incision should be larger than usual, considering the difficulty in securing the field of vision owing to body movements. It is also important to administer local xylocaine injections immediately at the first sign of pain, such as physical movement. As local anesthesia does not utilize muscle relaxants, monopolar devices should not be used because of unwanted muscle movements caused by electric currents or sudden body movements, which can be dangerous. However, energy devices effectively control bleeding, and the aggressive use of bipolar and vessel-sealing systems can control bleeding and shorten the operative time [7]. Atropine sulfate can be administered to reduce salivation and decrease swallowing, which is another technique used in surgery under regional anesthesia.

## Conclusions

We herein report a case of thyroid lobectomy performed under local anesthesia. Low-risk patients can reportedly be safely operated upon under local anesthesia, but caution should be exercised in high-risk patients. In such cases, it is imperative to minimize as much discomfort due to pain and dyspnea as possible.
